# MiR-221 Expression Level Correlates with Insulin-Induced Doxorubicin Resistance in MCF-7 Breast Cancer Cells

**DOI:** 10.22074/cellj.2021.7153

**Published:** 2021-07-17

**Authors:** Parisa Kheradmand, Sadeq Vallian Boroojeni, Saeed Esmaeili-Mahani

**Affiliations:** 1 Department of Cellular and Molecular Biology and Microbology, Faculty of Science and Technology, University of Isfahan, Isfahan, Iran; 2 Department of Biology, Shahid Bahonar University of Kerman, Kerman, Iran

**Keywords:** Breast Cancer, Doxorubicin, Insulin Receptor, MCF-7 Cells, MiR-221

## Abstract

**Objective:**

Insulin induces anti-cancer drugs resistance in tumor cells. However, the mechanism by which insulin induces its drug resistance effects is not clear. In the present study, the expression of miR-221 in insulin-treated MCF-7 cells in response to the anti-cancer drug doxorubicin, was investigated.

**Materials and Methods:**

In this experimental study, cell viability was evaluated using MTT (3-[4,5 dimethylthiazol-2- yl]-2,5-diphenyl tetrazolium bromide) assay. The expression level of miR-221 was determined by real time polymerase chain reaction (RT-PCR). Furthermore, the expression of insulin receptor (IR) and cleaved caspase-3 protein was assessed by Western blotting.

**Results:**

The results showed that treatment of the MCF-7 cells with insulin reduced the anti-cancer effects of doxorubicin. Viability of naive and insulin-treated cells following doxorubicin (DOX) treatment was 62.9 ± 5.7% and 79 ± 7.2%, respectively. Furthermore, the expression of miR-221 in insulin-treated cells was significantly increased (2.6 ± 0.37-fold change) as compared with the control group. A significant decrease (26%) in the expression of caspase-3 protein and a significant increase (24%) in IR were observed in insulin-induced drug resistant MCF-7 cells as compared to the naive cells.

**Conclusion:**

Together, the data showed a positive correlation between the expression of miR-221 and IR expression, but a negative correlation with caspase3 expression, in insulin-induced drug resistant MCF-7 breast cancer cells. This could suggest a new mechanism for the role of miR-221 in cancer drugs resistance induced by insulin.

## Introduction

Chemotherapy based on doxorubicin (DOX) is one of the most common treatments for breast cancer. DOX belongs to the family of anthracyclines, which functions through two main mechanisms: i. It can intercalate itself into the DNA and inhibit DNA and RNA polymerases and disrupt DNA repair mechanism by topoisomerase enzymes, and ii. DOX can cause the formation of free radicals resulting in damages to proteins, cell membranes and DNA. Despite advances in cancer therapy, drug resistance such as DOX resistance is one of the most important challenges.

Numerous studies showed the association between insulin signaling and tumor metastasis and drug resistance (1, 2). Due to its connection with a network of signaling pathways, insulin signaling has been considered one of the very complicated pathways (2). Upon binding of insulin to the α-subunit of insulin receptor (IR), conformational changes induce trans-phosphorylation of each β-subunit, resulting in the activation of IR. Subsequently, the activated IR phosphorylates intracellular substrates such as the IR substrate (IRS) family. IRS phosphorylation finally results in the activation of downstream effectors such as AKT (protein kinase B), which mediates several functions that prevent cell death and result in cell survival like activating protein and glycogen synthesis (2, 3). This cascade of phosphorylation events is commonly known as the PI3K/AKT pathway of insulin signaling which sometimes increases carcinogenicity (3, 4) and induces drug resistance (1, 5, 6). In fact, in many types of cancers, insulin induces resistance to chemotherapy and may even be associated with late diagnosis, especially in patients with obesity and type-2 diabetes.

A significant association between cancer-related mortality and use of exogenous insulin was reported (7). Moreover, the relevance of increased risk of breast cancer and type-2 diabetes in women was demonstrated (8). Furthermore, the association between diabetes and an increased risk of colorectal cancer was reported. In addition, it was reported that the up-regulation of IR can enhance multistage tumor progression and cause intrinsic resistance to insulin-like growth factor-1 receptor (IGF-1R) targeted therapy (9). Moreover, the over-expression of IRs in cancers was shown in different reports (9, 10). However, the mechanism(s) by which insulin induces drug resistance is not fully understood.

miRNAs are small noncoding RNAs (18-23 nucleotide) which are transcribed by RNA polymerase II and play critical roles in gene regulation (11). Recent studies indicated that more than half of the known human genes are targets for miRNAs and each miRNA can regulate multiple target genes (12). It is believed that more than 50% of miRNAs are located in the genomic regions that were deleted or duplicated in various types of tumors, leading to under regulation of gene expression (13). It was reported that up- or down-regulation of miRNAs expression could lead to variations in chemotherapy susceptibility of cancer cells through various cellular pathways (14, 15). Moreover, it was shown that several miRNAs can regulate cellular response to anti-cancer drugs by modifying drug concentration, survival pathway, apoptotic response and cell cycle (16). It was demonstrated that there is an aberrant expression of miRNAs such as miR-221, miR-21, miR-19, and miR-127, in drug-resistant cancer cells (17-19). Moreover, several reports indicate the involvement of miRNAs such as, miR-221, miR-181b, miR-126 and miR-21, in regulation of expression of genes involved in insulin signal transduction pathway (20, 21).

In this study, the changes in the expression of miR-221, IR and apoptotic components of caspase-3, were evaluated in insulin-induced drug resistant MCF-7 breast cancer cells.

## Materials and Methods

In this experimental study, cell culture reagents, fetal bovine serum (FBS), penicillin-streptomycin solution and trypsin-EDTA, were obtained from Biosera Company (Boussens, France). Cell culture flasks and dishes were purchased from SPL Life Science, Inc. (Gyeonggi-Do, South Korea). MTT (3-[4, 5-dimethyl-2-thiazolyl]-2, 5-diphenyl-2-tetrazolium bromide) and primary monoclonal anti-β-actin antibody (A-5316) were obtained from Sigma-Aldrich (St. Louis, MO). Primary polyclonal anti-Insulin Rβ (sc-711), secondary goat anti-rabbit (sc-2004), and secondary goat anti-mouse (sc- 2357) antibodies were purchased from Santa Cruz Biotechnology, Inc. (Santa Cruz, CA). Primary polyclonal anti-Caspase-3 (#9662) was purchased from Cell Signaling Technology (Danvers, MA, USA).

The present work was approved by Department of Research and Technology of University of Isfahan as a Ph.D. thesis. 

### Cell culture

The MCF-7 cell line was obtained from the National Cell Bank of Iran (Pasteur Institute, Tehran, Iran). The cells were cultured in Dulbecco’s modified Eagle’s medium (DMEM, Biosera, France) supplemented with 10% FBS, penicillin (100 U/mL), and streptomycin (100 μg/mL). They were maintained in 5% CO_2_ atmosphere at 37˚C. Cells were cultured in 96-well culture plates at initial seeding number of 5×10^3^ cells per well.

### Cell viability analysis

Cell viability was assessed by MTT assay (22). In this method, MTT is reduced to purple formazan by mitochondrial dehydrogenase, revealing the number of living cells. MTT was dissolved in phosphate-buffered saline (PBS) at final 5 mg/ml concentration. In each assay, 20 μl of MTT was added to each well containing 5×10^3^ MCF-7 cells, and then, incubated for 2 hours at 37˚C. In the next step, the culture medium was removed carefully and 100 μl dimethyl sulfoxide (DMSO) was added to the cells. The cell plate was gently shaken until formazan crystals were dissolved completely. Absorbance (optical density) was determined at 490 nm by an automatic microplate reader (ELX 8000, Biotek-USA).

In order to determine the antitumor effects of DOX in breast cancer cells, the MCF-7 cells were treated with the increasing concentrations of DOX and their viability was determined by MTT assay. After the initial 24 hours of attachment/growth period, the cells were incubated with DOX 1, 5 and 10 μM for 48 and 72 hours. The main studied groups in MTT test were naive cell group and insulin-treated cell group. The naive cells received no insulin treatment. This group included the following subgroups: i. Control cells which were cultured in 200 μl complete DMEM growth medium and ii. Three groups of MCF-7 cells that were incubated with different doses of DOX (1, 5 and 10 μM).

Treated MCF-7 cells group contained cells which were treated with insulin (48 or 72 hours). This group contained subgroups including control group, which was cultured in 200 μl complete DMEM growth medium with 10 nM insulin, and three groups of insulin pretreated MCF-7 cells that were incubated in the presence of different doses of DOX (1, 5 and 10 μM) for further 24 hours.

### Total RNA isolation and real time polymerase chain reaction

Total RNA extraction was performed from collected cells using RNX+ reagent (SinaClone Co., Iran), and then, cDNA was synthesized using a universal cDNA synthesis kit (Exiqon, Copenhagen, Denmark) according to the manufacturer’s protocol. U48 small nuclear RNA was used as the internal control. The real-time polymerase chain reaction (PCR) reactions were performed using the specific primers of hsa-miR-221 and U48 (Pars Genome co., Iran). Quantitative PCR (qPCR) was performed using 7500 real-time PCR system (Applied Biosystem-USA). In our experiments for comparing gene expression levels among samples, the 2^−ΔΔCT^ method was used (23). The main studied groups for RT-PCR were naive cells group and insulin-treated cells group. In naive cells group, no insulin treatment was done and this group included: control cells which were cultured in 200 μl complete DMEM growth medium and a group of MCF-7 cells that was incubated with DOX (10 μM). 

Insulin-treated cells group contained MCF-7 cells which were treated with insulin. This group contained a control group which was cultured in 200 μl complete DMEM growth medium supplemented with 10 nM insulin, and a group of insulin-pretreated MCF-7 cells that were incubated with DOX (10 μM) for 24 hours.

### Western blot analysis

Changes in the expression of caspases could significantly affect resistance to chemotherapy drugs (24, 25). In our study, we assessed the activated caspase-3 (as executive caspases) and IR protein expression, by western blotting.

MCF-7 cells were lysed and homogenized in ice-cold buffer containing 10 mM Tris-HCl (pH=7.4), 0.1% sodium dodecyl sulfate (SDS), 1 mM EDTA, 0.1% Na-deoxycholate, 1% NP-40 with protease inhibitors (1 mM phenylmethylsulfonyl fluoride, 2.5 μg/ml leupeptin, and 10 μg/ml aprotinin), and 1 mM sodium orthovanadate. The total proteins were extracted by centrifugation at 14,000×g for 15 minutes at 4˚C. Equal amounts of proteins (40 μg) were fractionated by sodium dodecyl sulfate polyacrylamide gel electrophoresis (9% SDS-PAGE) and transferred to polyvinylidenedifluoride (PVDF) membrane (Roche Co, Germany). After blocking at room temperature for 1 hour, the membranes were immunostained with primary antibodies against human IR (dilution, 1:1,000; sc-711; Santa Cruz Biotechnology, Inc., Dallas, TX, USA) and cleaved caspase-3 (1:1000 dilution, cell signaling, USA) at 4˚C, overnight. After washing, the membranes were incubated with matched horseradish peroxidase-conjugated secondary antibodies (1:10,000; Santa Cruz Biotechnology, Inc.) at room temperature for 1 hour. Then, the blots were assessed using the ECL system and imaged by Chemi Doc XRS+ imaging system (Bio-Rad Company, USA). The intensity of the bands was determined by Lab Works analyzing software (UVP, UK). In our immunoblot experiments, β-actin (1:10,000) was used as the loading control. Immune detection was recorded using Chemi Doc XRS+ imaging system (Bio-Rad Company, USA).

### Statistical analysis

All tests were performed in triplicate and the data was analyzed using SPSS (version 20) software (IBM, New York, NY, USA). The results are presented as mean ± standard error of the mean. For evaluating the differences in mean values among experimental groups, one-way analysis of variance was performed and it was followed by the Tukey test. A P<0.05 was considered significant.

## Results

### The effects of doxorubicin on naive and insulin-treated MCF-7 cells viability

**Fig.1:**
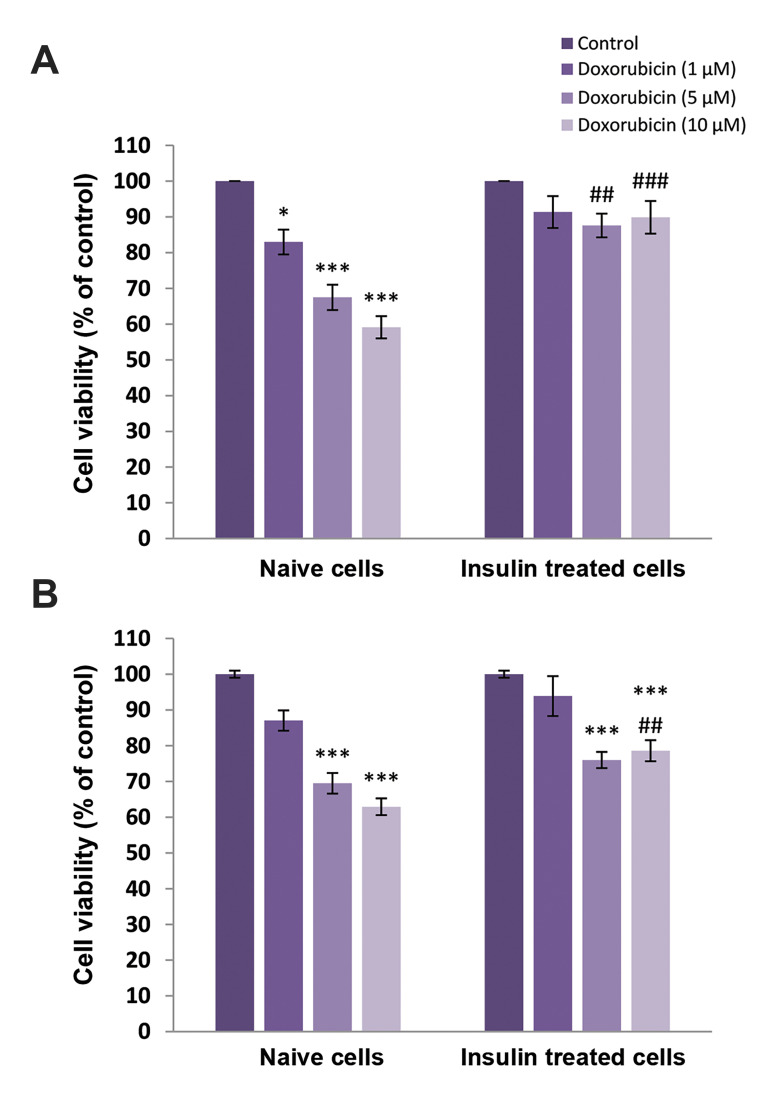
Effects of different concentrations of doxorubicin on naive and insulin-treated MCF-7 cells viability. The cells were pretreated with insulin 10 nΜ and vehicle for **A. **48 and **B.** 72 hours. Cell viability was determined by MTT assay. Data is expressed as mean ± SEM (n=6 wells for each group). ^*;^ P<0.05, ^***;^ P<0.001 are significantly different versus the control group, ^##^; P<0.01, and ^###^; P<0.001 are significantly different versus naive cells at the same dose of doxorubicin.

As shown in Figure 1, DOX had antitumor effects on MCF-7 cells in a dose-dependent manner. A significant effect was observed in the cells treated with 5 (P<0.05) and 10 μM (P<0.001) DOX (indicated as naive cells in Fig.1). Furthermore, to examine the effects of insulin treatment on doxorubicin-induced tumor cell death, distinct group of cells were pretreated with 10 nM insulin for 48 (Fig.1A) or 72 hours (Fig.1B) and then, different doses of DOX were added for an additional 24 hours. Our data showed that insulin could induce DOX resistance in MCF-7 cells.

### MiR-221 expression in naive and insulin-treated cells 

**Fig.2:**
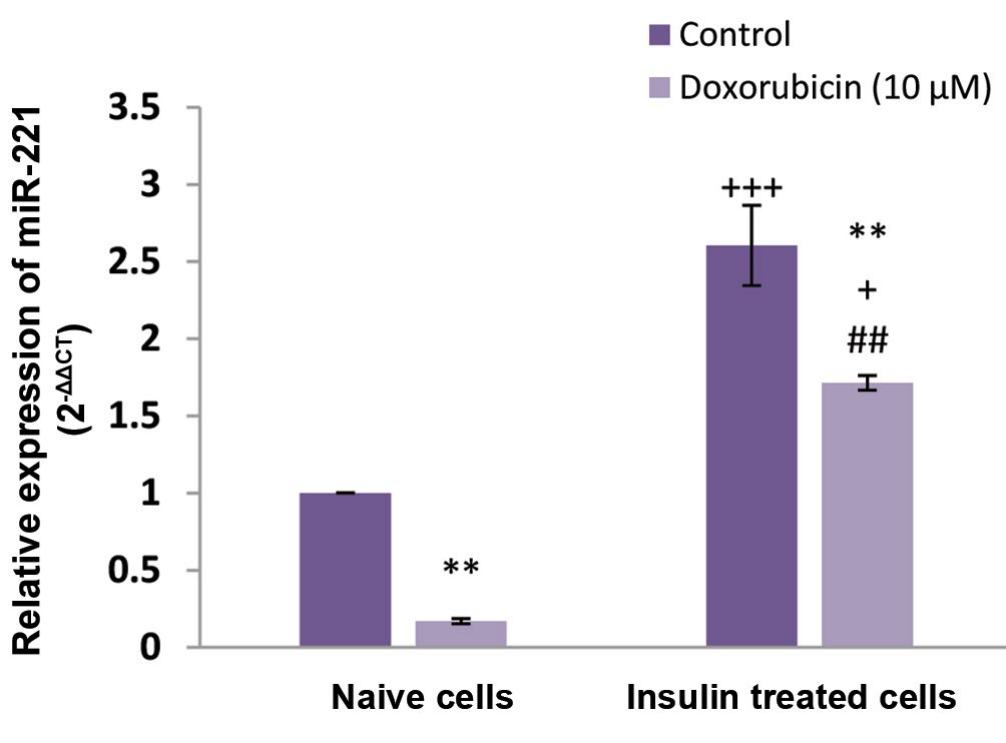
The effects of insulin treatment on miR-221 expression in naive (control) and insulin-pretreated MCF-7 cells in the presence of doxorubicin (10 μM) or vehicle. **; P<0.01 is significantly different versus control group, +; P<0.05, +++; P<0.001 are significantly different versus control naive cells, and ##; P<0.01 is significantly different versus doxorubicin-treated naive cells. The data was analyzed by 2^-ΔΔCt^.

To investigate the changes in miR-221 expression following the development of DOX resistance, the expression level of miR-221 was evaluated by qRT-PCR. As shown in Figure 2, miR-221 expression was up-regulated in insulin-treated MCF-7 cells. DOX could significantly decrease miR-221 levels in naive and insulin-treated cells. However, in the presence of doxorubicin, the data showed that the level of miR-221 in insulin-pretreated cells was greater than those in naive cells (Fig.2, Table 1).

As indicated in Figures 1 and 2, MiR-221 expression was evaluated in different situations including control MCF-7 naive cells, DOX- treated MCF-7 naive cells, insulin-treated MCF-7 cells and insulin + DOX-treated MCF-7 cells.

**Table 1:** Expression of miR-221 in naive (control) and insulin-pretreated MCF-7 cells in the presence of doxorubicin (10 μM) or vehicle


naive cell		Insulin-treated cell	
Control	Doxorubicin	Insulin	Doxorubicin+Insulin

1	0.17 ± 0.03	2.6 ± 0.37	1.71 ± 0.07


### The expression level of caspase-3 protein in naive and insulin-treated MCF-7 cells

**Fig.3:**
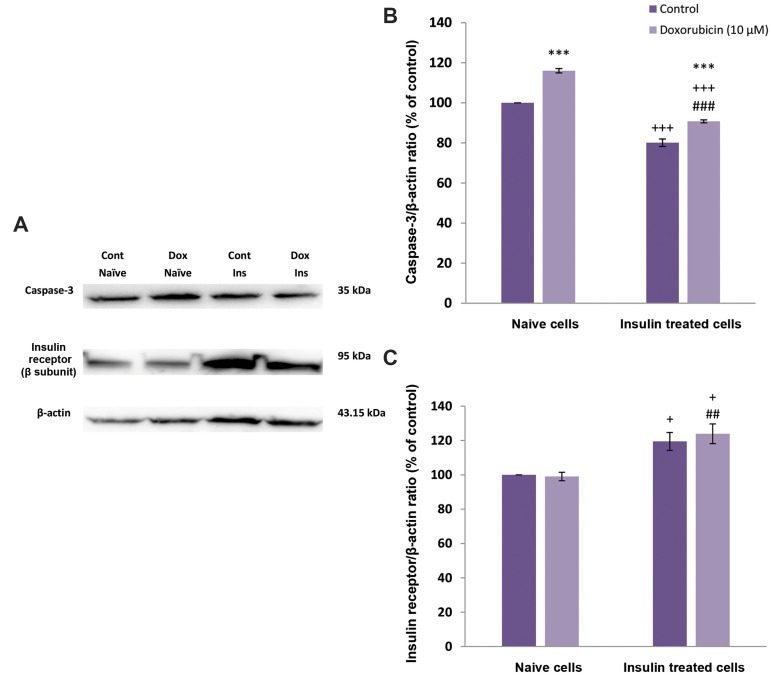
The effects of insulin treatment on caspase-3 and insulin receptor protein levels in naive (control) and insulin-pretreated MCF-7 cells in the presence of doxorubicin (10 μM) or vehicle. **A.** Protein bands were detected by Western blot analysis. **B. **Ratio of caspase-3 to β-actin level.** C. **Ratio of insulin receptor to β-actin level. β-actin was used as an internal control. Each value in the graph represents mean ± SEM band density ratio for each group. ***; P<0.001 is significantly different versus control group, +++; P<0.001 is significantly different versus control naive cells, ###; P<0.001 is significantly different versus doxorubicin-treated naive cells, +; P<0.05 is significantly different versus the control naive cells, ##; P<0.01 is significantly different versus the doxorubicin (DOX)-treated naive cells, Cont; Control, Dox; Doxorubicin, and Ins; Insulin.

The expression level of activated caspase-3 protein was investigated by Western blotting in naive and insulin-treated MCF-7 cells. The data showed that incubation with 10 μM DOX could significantly increase caspase-3 band density in naive cells. However, in the presence of insulin, the expression of caspase-3 was reduced in comparison with the naive cells (Fig.3A, B).

### The expression level of insulin receptor (subunit β) protein in naive and insulin-treated MCF-7 cells

To examine the contribution of changes in IR protein density, Western blotting was used to evaluate the level of expression of IR in naive and insulin-treated MCF-7 cells. The results showed that treatment with 10 nM insulin could significantly increase IR band density (Fig.3A, C).

## Discussion

Drug resistance especially to DOX (a commonly used drug), is a major obstacle for breast cancer chemotherapy. Different genes were found to be associated with DOX resistance. Reduced expression of cyclin D2, cyclin B1 and p-ERK1 were shown to cause DOX resistance in breast cancer cell lines (26). Furthermore, decreased expression of miR-298 was found to be significantly correlated with DOX resistance in MDA-MB-231 cells (27). 

The results of this study clearly showed that insulin can cause DOX resistance in MCF-7 breast cancer cell lines. The data suggested that induction of DOX resistance by insulin might be through i. Overexpression of miR-221, ii. Increases in the expression of IR, and iii. Down regulation of caspase-3. 

In several studies, a significant association between the risk of cancer and use of exogenous insulin or up regulation of IR was reported (28). Furthermore, it was shown that insulin can cause drug resistance in different types of cancer (29). However, the detailed mechanism (s) has not been fully clarified.

In this study, the data showed that insulin treatment can lead to DOX resistance. Previous studies showed that breast cancer cells were not able to reduce IR sensitivity in the presence of high doses of insulin (30). The overexpression of IR in insulin-treated MCF-7 cells resulted in an increase in insulin signal transduction. It was documented that insulin through its tyrosine kinase receptor, can control proliferation, differentiation, and survival of cells via two signaling pathways including PI3K/AKT and Ras-MAPK (5).

Different studies demonstrated that increased activity of PI3K/AKT pathway is associated with cancer progression, invasion, epithelial-mesenchymal transition and resistance to anti-cancer drugs (28-30). PI3K/AKT signaling pathway is a complex signaling network that can regulate several proteins by multiple mechanisms of regulation. For example, PI3K/AKT activation can phosphorylate glycogen synthase kinase 3 β (GSK-3β), which suppresses GSK-3β (31). This process leads to stabilization of nuclear β-catenin followed by transactivation of slug transcription factor (32). Recent studies showed that slug, a repressor of E-cadherin, has an important role in the epithelial-mesenchymal transition in cancer cells (33). It was reported that miR-221 expression is related to slug as a transcription factor (34) suggesting that over expression of miR-221 in insulin-treated MCF-7 cells may partially result from slug over expression which was induced by the activation of PI3K/AKT signaling. As these studies showed, slug transcription factor silencing by siRNA against slug could significantly decrease miR-221 expression. This finding may lead to the development of therapeutic strategies for overcoming insulin-induced drug resistance in breast cancer. 

Recently, several studies indicated that miR-221 has an important role in repressing the expression of caspase-3 as its target gene (24, 25). Moreover, it was indicated that p53, as a tumor suppressor, could play a critical role in tumor cells apoptosis (35, 36) and its activation is one of the important mechanism of antitumor drugs (37, 38). It was demonstrated that DOX induced apoptosis in MCF-7 cells through p53 activation followed by caspase-3 activation and DNA fragmentation (39). Using the MTT assay, we found that DOX reduced viability of naive MCF-7 cells. On the other hand, insulin pretreatment before DOX incubation, could increase viability of MCF-7 cells in comparison with naive DOX-treated cells, and therefore, caused DOX resistance. Our results, as confirmed by Western blotting assay, showed that caspase-3 expression level increased under DOX treatment in naive cells, and its expression level decreased after insulin treatment. This suggested that miR-221 overexpression through insulin treatment could led to caspase-3 down regulation. In a collaborative project with Dr. Haddadi, in Department of Biology, Shahid Bahonar University of Kerman, the expression of protein levels of Bax and Bcl-2 is under investigation (personal communication, unpublished data). Their preliminary data indicated that insulin induced drug resistance by increasing Bcl-2/Bax ratio and prevention of apoptosis in MCF7 cells.

Taken together, the present data suggested that insulin could induce DOX resistance in breast cancer cells. This happens through, at least in part, miR-221 overexpression as one of the key regulator of both PI3K/AKT, and Ras-MAPK in insulin signaling pathway followed by caspase-3 down regulation. Meanwhile, it would be interesting to investigate, in a new study, the expression of other molecules involved in this signaling pathway. Our observations could help clarifying one of the possible mechanisms of insulin-induced drug resistance in MCF-7 as a well-known breast cancer cell line. Nevertheless, performing the experiments in other cells would be part of our future projects to elucidate the mechanisms by which insulin affects breast cancer drug resistance. 

## Conclusion

In the present work, the expression of miR-221 in insulin-treated MCF-7 cells in response to anti-cancer drug DOX was investigated. Furthermore, the expression of cleaved caspase-3 protein and IR was examined. The main findings of this research could be summarized as follows: i. In the presence of DOX, the miR-221 expression level in insulin-pretreated cells was greater than those in naive cells, ii. DOX incubation could significantly increase caspase-3 band density in naive cells. However, in the presence of insulin, the expression of caspase-3 was reduced in comparison with the naive cells, and iii. Insulin treatment could significantly increase IR band density in insulin-treated cells.
